# Video Presentation of Myokymia in a Snakebite

**DOI:** 10.7759/cureus.16844

**Published:** 2021-08-02

**Authors:** Aman Garg, Baldeep Kaur, Yuvraj S Cheema, Shivani Gupta, Tagru Raju

**Affiliations:** 1 General Medicine, Government Medical College and Hospital, Chandigarh, Chandigarh, IND

**Keywords:** snakebite, myokymia, involuntary, venom, fasciculation

## Abstract

Patients with snakebites have highly variable presentations, and delayed diagnosis may lead to unfavorable outcomes. Here, we describe the case of a snakebite in a 23-year-old male who presented with myokymias. On management with mechanical ventilation and anti-snake venom, the patient improved and was discharged. The presence of myokymias may be an early clue to diagnosis and the need for mechanical ventilation in a patient with a snakebite.

## Introduction

Snakebite is an important public health problem in many tropical and subtropical countries. Globally, at least 2.7 million envenomings and 80,000 to 138,000 deaths occur annually due to snakebite [1]. Clinical presentations can be variable depending upon the geographical location and the species of the snake. Systemic features include clotting defects, neurotoxicity, myotoxicity, cardiotoxicity, and nephrotoxicity [2]. Myokymia is a rare presentation of neurotoxic snakebite, characterized by spontaneous, fine, involuntary, undulating ripples of muscle fibers [3]. It has earlier been reported as a rare presentation of a bite from timber rattlesnake (*Crotalus horridus*) in the United States [4]. Here, we present a case of myokymia that resolved after two days of anti-snake venom therapy in India.

## Case presentation

A 23-year-old farmer was brought to casualty in a state of drowsiness with a brief history of limb weakness and difficulty in swallowing and speaking. Attendants stated that he witnessed a snake at his workplace but did not know if he was bitten. He had fairly stable vitals and a Glasgow Coma Scale score of 15. His pupils were mid-dilated and reacted normally to light. Bulbar weakness was present. The single breath count was nil as the patient was unable to speak. Diffuse involuntary undulating waves of muscle fibers likely myokymia were seen in upper and lower limbs bilaterally, as shown in Video 1.

**Video 1:** Video showing involuntary undulating waves of muscle fibers suggestive of myokymia in a patient with snakebite.

The patient subsequently became unconscious within a few minutes and developed respiratory paralysis. He was intubated and started on mechanical ventilation. Snakebite was suspected according to history and clinical findings. He was administered stat intravenous dose of injection neostigmine 1.5 mg, followed by 0.5 mg every 30 minutes and injection atropine 0.6 mg intravenous every half-hourly. A total of 20 vials of polyvalent snake antivenom were administered over six hours as per the Government of India guidelines. Myokymias reduced significantly on the same day and disappeared completely over the next two days. The patient was weaned off the ventilator and subsequently discharged after full recovery.

## Discussion

Neurotoxic snakebites usually present as ptosis and external ophthalmoplegia, which can appear from minutes to hours after the bite. Subsequently, muscles of the face, neck, and deglutination become paralyzed. The involvement of intercostal muscles and diaphragm leads to respiratory failure [5]. Hemostatic abnormalities present as prolonged bleeding from a fang puncture wound or venipuncture site, epistaxis, hematemesis, hemoptysis, cutaneous ecchymosis, subconjunctival, retroperitoneal, or intracranial hemorrhage. Vipers and sea snakes may also cause intravascular hemolysis [2]. Myokymia, though not well defined in all snakebites, is a unique neurotoxic symptom previously reported in timber rattlesnake [4]. It is an involuntary vermicular movement of the muscles visualized over the skin as continuous ripples. It is difficult to distinguish from fasciculations which occur as individual muscle twitches. Myokymia is a chain of short-lived axonal bursts of a single motor unit firing at a rate of 5-150 Hz, with each burst occurring as a doublet or multiplet. Although fasciculations involve a single motor unit, they do not occur in cyclic bursts [3].

Interaction of snake venom with calcium or voltage-gated potassium channels in the nerve axons leading to modification in the axonal microenvironment may be the possible cause of myokymic discharge [3,4,6].

A case report from San Fernando described a 41-year-old male bitten by a pit viper who developed abdominal and limb pain along with diffuse myokymias in the thigh muscles. Electromyogram revealed neuronal hyperexcitability with mild myopathic features, while the electroencephalogram showed increased cortical irritability in the left parietal and temporal area. The authors described myokymia as a warning sign for mechanical ventilation, which was also required in our patient [7].

Another case report from Arizona described a 14-year-old boy bitten by a rattlesnake who presented with generalized myokymia over the face and limbs, with no significant coagulopathy, which resolved with 10 vials of antivenom. A 40-year-old woman bitten by a rattlesnake developed perioral paresthesias and myokymia, which resolved promptly with calcium chloride (2 g of 10%) indicating the role of snake venom and calcium channel interaction, as described above. Her clinical course was remarkable for local swelling, rhabdomyolysis, and mild hypofibrinogenemia for which she received 30 vials of polyvalent antivenom (*Crotalidae*). She was discharged later following marked clinical improvement [8].

## Conclusions

At present, it is difficult to establish the exact cause of myokymia in snakebites. Further studies are required to identify other groups of snakebites that present with myokymia, how many patients with myokymia require mechanical ventilation, and whether they are responsible for rhabdomyolysis. However, early recognition is crucial as myokymia can be the first warning sign for mechanical ventilation and is easily reversed by snake antivenom.
